# An ultrasensitive electrochemical sensing platform for the detection of cTnI based on aptamer recognition and signal amplification assisted by TdT

**DOI:** 10.1039/d0ra05171c

**Published:** 2020-10-05

**Authors:** Mingjian Lang, Dan Luo, Guangyi Yang, Quanxi Mei, Guangjun Feng, Yang Yang, Zhaohui Liu, Qinhua Chen, Lun Wu

**Affiliations:** The Fifth People's Hospital Affiliated to Chengdu University of Traditional Chinese Medicine 611130 Chengdu Sichuan China; Shenzhen Baoan Authentic TCM Therapy Hospital 518101 Shenzhen Guangdong China cqh77@163.com; Affiliated Dongfeng Hospital, Hubei University of Medicine 442008S Shiyan Hubei China wulun0909@163.com

## Abstract

We have developed an ultrasensitive and highly specific electrochemical sensing platform for the detection of cardiac troponin I (cTnI), a recognized biomarker for the diagnosis of acute myocardial infarction (AMI) and related cardiovascular diseases (CVDs). This strategy is based on the assists of terminal deoxynucleotidyl transferase (TdT)-mediated signal amplification and the specific recognition between cTnI and the aptamer of cTnI. In this experiment, we prepared a gold electrode that modified with probe 2 (P2), in the presence of cTnI, the aptamer of cTnI that in probe 1 (P1)/aptamer complexes bond with cTnI specifically and release the free P1. P1 would bind with P2, resulting in the formation of 3′-OH of DNA. In the presence of terminal deoxynucleotidyl transferase (TdT) and dTTP, TdT mediated P1 to extend and formed the structure of poly T. Methylene blue (MB)-poly A hybridized with the extended poly T and generated an electrochemical signal. The detection limit can be as low as 40 pg mL^−1^. This sensor was also successfully applied to the detection of cTnI in numerous spiked biological samples, and it can be a great reference for the clinical diagnosis, prognosis, and treatment of CVDs and AMI.

## Abstract 2

We have developed an ultrasensitive and highly specific electrochemical sensing platform for the detection of cardiac troponin I (cTnI), a recognized biomarker for the diagnosis of acute myocardial infarction (AMI) and related cardiovascular diseases (CVDs).

## Graphical abstract

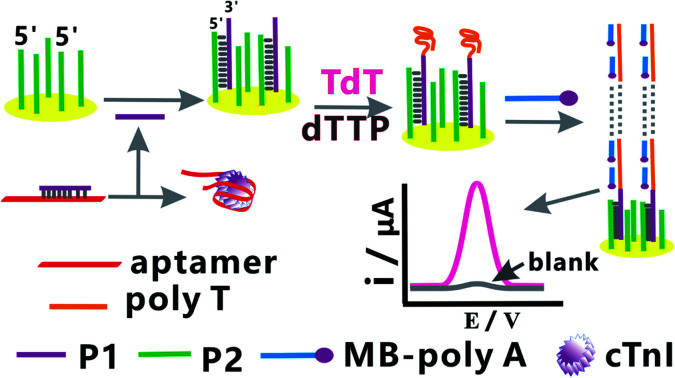

## 1. Introduction

Cardiovascular diseases (CVDs) are major causes of death in the world, causing serious problems to human health.^1,2^ The rates of morbidity and mortality due to CVDs are expected to continue to rise rapidly with the aging of the population in the future.^3^ Acute myocardial infarction (AMI) is a common cardiovascular disease; it is also one of the most serious adverse cardiac events, which may lead to irreversible myocardial injury or necrosis.^4^ At present, the diagnosis of AMI is still based on electrocardiography (ECG), but only a small proportion of patients with AMI show changes in ECG and can be diagnosed.^5,6^ Therefore, we need a timely way that can assist the early diagnosis and treatment of the patients with AMI and improve their survival rate. Clinicians often use biomarkers to assist in the assessment of AMI.^7^ Since the redefinition of the diagnostic standard of AMI in 2000, cardiac troponins (cTns) have been regarded as better biomarkers in the diagnosis of AMI.^8^ The recent related literature reports on CVDs and AMI strongly prefer cardiac troponin I (cTnI) as the biomarker^9^ of choice due to its sensitivity and specificity in the diagnosis of CVDs and AMI.^8,10,11^

cTnI is a polypeptide involved in myocardial contraction,^12^ which is released only during cardiomyocyte necrosis,^13^ and it is not found in smooth muscle nor is it found in substantial concentrations in striated muscle, showing little or no changes in patients with a skeletal muscle disease or trauma.^14^ Thus, at present, cTnI is widely used as one of the reliable biomarkers of AMI due to its high specificity and high sensitivity,^12,15–17^ and it is gradually becoming the “gold standard” in clinical practice for the diagnosis of AMI.^13,18,19^ cTnI may also be used to monitor the state of patients with AMI and predict the prognosis.^20^ In the previously related literature, several techniques have been reported to monitor cTnI, including the enzyme-linked immunosorbent assay (ELISA),^21^ fluoro-microbead guiding chip,^14^ surface plasmon resonance (SPR) biosensors,^18^ silicon nanowire biosensors,^21^ electrochemiluminescence immunosensors,^22^ nanoelectrode arrays,^23^ fluorescence probes,^19^ radioimmunoassay (RIA),^24^ and optomagnetic biosensors.^25^ However, all of the above methods have their own disadvantages: ELISA requires a long time to operate and a well-trained operator;^21^ the other biosensor methods for cTnI detection are usually complex in process or operation, labor-intensive, time-consuming, and involve expensive reagents.^14,18–25^ Therefore, we need to develop a convenient and highly specific method for the detection of cTnI, which can assist the diagnosis and treatment of CVDs and AMI.

Nowadays, electrochemical biosensors have attracted particular attention as a powerful diagnostic platform due to their remarkable advantages of being ultrasensitive, highly selective, simple, fast, portable, real-time, and inexpensive for sensing applications.^26–28^ In this study, we have designed a novel, highly specific, and ultrasensitive electrochemical biosensor for the signal amplification and detection of cTnI, which can assist the clinical diagnosis of CVDs and AMI. In this study, first, we achieved high selectivity through the specific binding of aptamers to the target cTnI. An aptamer is a small oligonucleotide sequence screened *in vitro*, which can bind with corresponding ligands with high affinity and strong specificity.^29^ Compared with antibodies, an aptamer has higher stability and can be easily synthesized. Its emergence provides a new research platform for biochemical and biomedical fields with high efficiency and fast recognition, and shows good application prospects in many aspects.^30^ Accordingly, the aptamer of cTnI can bind with cTnI specifically in our sensing process. Second, we used terminal deoxynucleotidyl transferase (TdT) to complete the amplification of the electrical signal and thus achieved the aim of ultrasensitive detection of cTnI in this electrochemical sensing platform. Terminal deoxynucleotidyl transferase (TdT), also known as terminal transferase,^31^ is a kind of DNA polymerase that can catalyze the sequential addition of deoxy-ribonucleoside triphosphates (dNTPs) at the 3′ hydroxyl (3′-OH group) terminus of the target DNA^32–35^ and extend the strand of DNA to form a polynucleotide tail (such as poly T) to advocate signal amplification.^36,37^ Deoxythymidine triphosphate (dTTP) is one of the dNTPs and is also one of the direct precursors of DNA biosynthesis.^35,38–40^ Finally, we used methylene blue (MB), an aromatic heterocycle^41,42^ that was modified with poly A, as an effective electroactive hybridization indicator^43–45^ in this electrochemical sensing platform for the detection of cTnI.

Hence, in the present research, we have developed an ultrasensitive and specific electrochemical biosensor that combined the signal amplification strategy based on TdT with specific recognition between the aptamer of cTnI and cTnI. The aptamer of cTnI specifically bonds with cTnI in the presence of cTnI, resulting in the release of free P1. The free P1 hybridized with P2 modified on the surface of the gold electrode and formed the strand of P1/P2. With the addition of TdT and dTTP, TdT extended the strand of P1 and formed the structure of poly T due to the presence of free –OH at the 3′ end of P1. The extended poly T hybridized with MB-poly A and thus introduced MB to the surface of the gold electrode, resulting in the formation of the electrochemical signal. One extended poly T can hybridize with multiple MB-poly A and lead to signal amplification. By detecting the amplified electrochemical signal, we can achieve the detection of low content cTnI. In addition, this highly specific and ultrasensitive electrochemical biosensor for the detection of cTnI is simple, novel, inexpensive, and fast. This study can accelerate the development of myocardial markers' determination, which provides a potential reference for the clinical diagnosis, prognosis, and treatment of CVDs and AMI.

## 2. Experimental

### 2.1. Materials

The five proteins (cTnI, myoglobin, EpCAM, CRP, and CEA) used in this study were purchased from Cusabio Biotechnology Co. Ltd (Wuhan, China). TdT and dTTP were purchased from Takara Biotech Co. Ltd (Dalian, China). Gold electrodes were obtained from Gaoss Union Technology Co. Ltd (Wuhan, China), and 6-mercapto-1-hexanol (MCH) was obtained from Sigma-Aldrich (St. Louis, MO, USA). All synthetic DNA sequences (P1, P2, MB-poly A, and the aptamer of cTnI) used in the experiments were obtained from Sangon Biotechnology Co. Ltd (Shanghai, China); their sequences are listed in Table 1. The buffer solution used in the experiments is 10 mM Tris (10 mM MgCl_2_, 50 mM NaCl). All other reagents used in the experiments are of analytical grade, and ultrapure water was obtained from the Millipore water purification system (18.2 MΩ cm resistivity, Milli-Q Direct 8). The biological samples, including the serum, saliva and urine, of healthy normal human subjects were all from Dongfeng General Hospital affiliated to Hubei University of Medicine. All experiments in this study were conducted in strict accordance with the institutional guidelines and relevant laws (National Health Commission of the People's Republic of China, ethical review of biomedical research involving human beings), and it has been approved by the Ethics Committee of Dongfeng General Hospital (Shiyan, China). Informed consent has been obtained from human subjects involved in any experiments.

**Table 1:** All synthetic DNA sequences and aptamer of cTnI used in this study

Name	Sequences
Aptamer of cTnI	5′-CGT GCA GTA CGC CAA CCT TTC TCA TGC GCT GCC CCT CTT A-3′
P1	3′-T CAT GCG GTT GGA AAG AG
P2	5′-CGC CAA CCT TTC TC-TTT-(CH_2_)_6_-SH
MB-poly A	MB-AAA AAA AAA AAA

### 2.2. Apparatus

In this study, square wave voltammetry (SWV) and chronoamperometry were performed on a CHI660D workstation (CH Instruments Inc., Shanghai, China). SWV was carried out in a 10 mM Tris buffer by scanning the potential from −0.4 to 0 V at an amplitude of 25 mV, a frequency of 25 Hz, and a step potential of 4 mV. Electrochemical impedance spectroscopy (EIS) and cyclic voltammetry (CV) experiments were performed in 10 mM phosphate-buffered saline (PBS) solution containing 0.1 M KCl and 5 mM [Fe(CN)_6_]^3−/4−^ in the potential window range of −0.2 to 0.6 V and the frequency range of 0.1 Hz to 10 kHz at a scanning rate of 0.1 V s^−1^ and amplitude of 5 mV.

### 2.3. Sensing process of cTnI

First, the aptamer of cTnI and P1 were added in equal proportion and hybridized, and then a certain concentration of cTnI antigen was added. 30 min later, 10 μL of the above solution was dropped onto the surface of the gold electrode that was modified with P2. After 15 min of reaction, the obtained gold electrode was immersed in the solution containing TdT and dTTP (2 mM). Then, the gold electrode was immersed in the MB-poly A (MB-AAA AAA AAA) solution, and 10 mM Tris buffer was used to wash after each step of the reaction. Finally, detect the electrochemical signal produced in the above process.

## 3. Results and discussion

### 3.1. Electrochemical characterization of the sensing platform

**Fig. 1:**
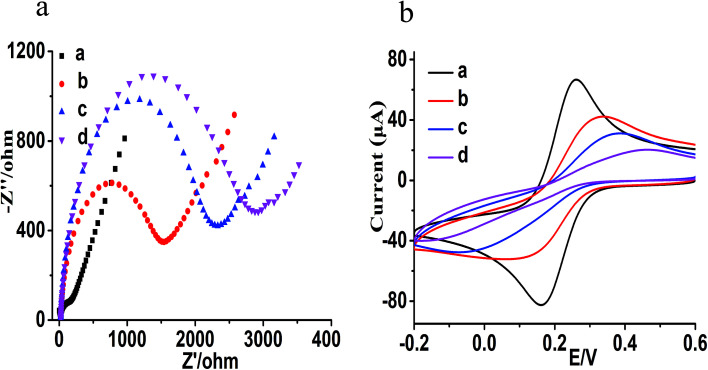
(a) The EIS characterization of the electrochemical sensing platform (a: the bare gold electrode; b: the electrode modified with P2 and sealed with MCH; c: TdT mediated extension; d: after hybridization of poly T and MB-poly A). (b) The CV characterization of the electrochemical sensing platform (a: the bare gold electrode; b: the electrode modified with P2 and sealed with MCH; c: TdT mediated extension; d: after hybridization of poly T and MB-poly A).

Prior to the experiments of this study, we conducted electrochemical impedance spectroscopy (EIS) and cyclic voltammetry (CV) to analyze the feasibility and construction of the electrochemical sensing platform; the results are shown in Fig. 1. EIS is usually used to investigate the modification process of the electrochemical sensors, and an increase in the semicircle diameter indicates an increase in the electron transfer resistance (*R*_et_) on the electrode surface. Additionally, CV is used to further characterize the construction process. As shown in Fig. 1a and b, the bare gold electrode presents a small semicircle area with an electron transfer *R*_et_ of 150 Ω (Fig. 1a, curve a) and shows the maximum redox peak current (Fig. 1b, curve a). After modifying the surface of gold electrode with P2 and sealing with 6-mercapto-1-hexanol (MCH), the semicircle diameter increased and the electron transfer *R*_et_ was 1500 Ω (Fig. 1a, curve b). The peak current decreased significantly, while the separation of the peak increased (Fig. 1b, curve b) in comparison with the bare gold electrode (Fig. 1b, curve a). It shows that P2 was modified on the surface of the bare gold electrode successfully, thus forming an Au–S bond *via* self-assembly. After adding cTnI aptamer/P1 and cTnI, P1 was released and hybridized with P2 (there was no significant change in the electrode). After adding TdT, TdT mediated P1 (because the 3′ end of P1 chain is free OH) to extend and obtain the structure of poly T, resulting in further increase in the semicircle with the *R*_et_ value of 2300 Ω (Fig. 1a, curve c) and further decrease in the peak current (Fig. 1b, curve c), which indicates the formation of P1/P2 and poly T. When the modified electrode was further hybridized with MB-poly A in the presence of P1/P2 and poly T, the semicircular diameter (*R*_et_ = 2900 Ω) further increased significantly (Fig. 1a, curve d), and the redox peak current further decreased (Fig. 1b, curve d). The above results show that the hybridization and extension of MB-poly A and poly-T triggered the signal amplification reaction that is based on the catalysis of TdT, and more MB bind to the surface of the electrode to form a stronger electrical signal.

### 3.2. Mechanism of cTnI electrochemical detection

**Fig. 2:**
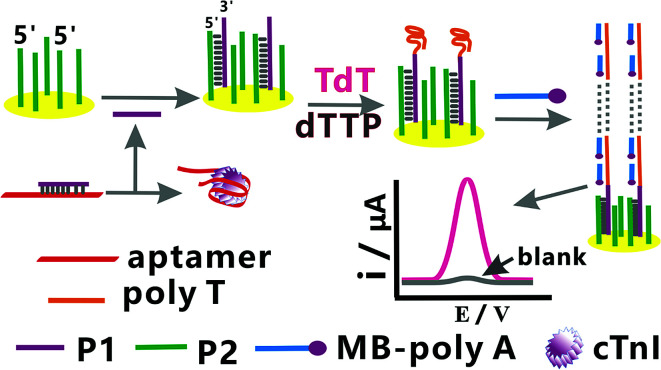
The schematic of this electrochemical sensing platform for the detection of cTnI.

Fig. 2 shows the principle of the electrochemical sensing platform for the detection of cTnI. Strand P1 can bind non-specifically with the aptamer of cTnI and form P1/aptamer complexes. With the addition of cTnI, the aptamer of cTnI in the P1/aptamer complexes would bind to cTnI specifically and release free P1. The free P1 would hybridize with P2 on the surface of the gold electrode and form the DNA hybridization double strand of P1/P2 on the electrode surface, resulting in the formation of 3′-OH of DNA. Because there is free –OH at the 3′ end of P1, in the presence of TdT and dTTP (one of the direct precursors of DNA biosynthesis), TdT would extend P1 to form the structure of poly T on the electrode surface and mediate signal amplification.^36,37^ The super long poly T can hybridize with multiple MB-poly A (MB-AAA AAA AAA), and thus introduce MB to the surface of the gold electrode. MB is an electron transfer mediator,^45^ and hence, the corresponding signal amplification can be observed. By detecting the amplified electrochemical signal in the aforementioned process, a highly sensitive and specific detection of cTnI can be achieved.

### 3.3. Optimization of experimental parameters

In order to obtain the best experimental performance of this electrochemical sensing platform, we used square wave voltammetry (SWV) to optimize the following experimental parameters: the concentration of P2, the concentration of cTnI aptamer, the amount of TdT and the reaction time. In each optimization experiment, the concentration of cTnI used was 10 ng mL^−1^, and each experiment was repeated three times.

**Fig. 3:**
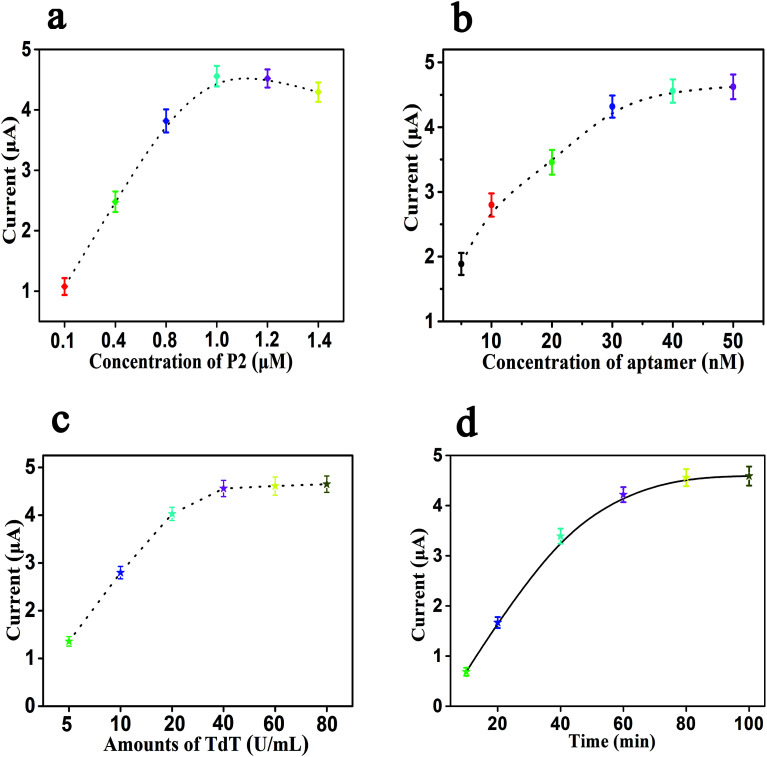
Effects of different conditions on the performance of cTnI electrochemical aptasensing platform. (a) Concentration of P2; (b) concentration of aptamer; (c) amounts of TdT; (d) reaction time; error bars: SD, *n* = 3.

The electrochemical signal of this sensor depends on the concentration of P2 fixed on the surface of the gold electrode, and too high or too low assembly concentration of P2 will make the electrode crowding and probe sinking, thus reducing the molecular recognition ability and the hybridization efficiency, and weakening the signal amplification performance. Therefore, first, we studied the effects of the concentration of P2 in the range of 0.1 to 1.4 μM on the current response of SWV. As shown in Fig. 3a, the current response increases gradually with the increase in the concentration of P2 from 0.1 μM to 1.0 μM, and then decreases gradually when the concentration of P2 exceeds 1.0 μM. The above results show that the optimal concentration of P2 is 1.0 μM, and it can be fixed on the electrode surface in a relatively vertical direction to obtain the best molecular recognition and hybridization efficiency. With the increase in the concentration of P2, the current response decreases gradually, which is due to the increase in the steric resistance and electrostatic repulsion, and it is unfavorable for surface hybridization. Consequently, the concentration of 1.0 μM for P2 was considered as the optimal concentration. The concentration of the cTnI aptamer also has an important effect on signal amplification, which may affect the efficiency of P1 to form the structure of poly T and trigger the electrochemical signal amplification in the presence of cTnI and TdT. As shown in Fig. 3b, the current response increases gradually with the increase in the concentration of the cTnI aptamer, and then it tends to be stable. When the concentration of the cTnI aptamer reaches 40 nM, the value of the current response also reaches a plateau. When the concentration of the cTnI aptamer is more than 40 nM, the corresponding change in the current response is negligible. Thus, we chose 40 nM as the optimal concentration of the cTnI aptamer.

Moreover, the amount of TdT is also one of the important factors that affected the efficiency of this biosensor, and TdT can mediate the extension of P1 to obtain the structure of poly T, which is the key part of the signal amplification of this sensing platform. The effect of the amount of TdT in this experiment is shown in Fig. 3c. From Fig. 3c, we can see that the value of the current response gradually increases with the increase in the amount of TdT (from 5 U mL^−1^ to 40 U mL^−1^); when the amount of TdT reaches 40 U mL^−1^, the value of the current response tends to be stable. When the amount of TdT is 60 U mL^−1^ or 80 U mL^−1^, the corresponding change in the current response can be ignored, so we selected 40 U mL^−1^ as the optimal amount of TdT that can obtain the maximum experimental signal. Finally, we also studied the effects of the reaction time, and the results are shown in Fig. 3d. It can be seen from Fig. 3d that the value of the current response increases gradually with the increase in the reaction time. When the reaction time is 80 min, the corresponding value of the current response reaches a relatively stable state. When the reaction time continues to increase to 100 min, the change in the current response value can be ignored. Therefore, 80 min is set as the optimal reaction time.

### 3.4. Sensitivity towards cTnI detection

**Fig. 4:**
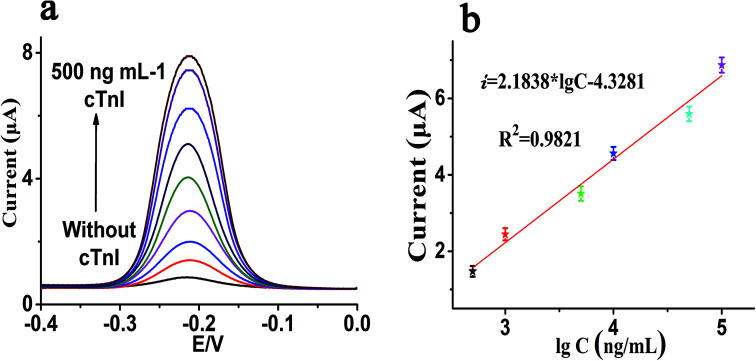
(a) Typical SWV current response of P2 modified electrode to a series of concentration of cTnI (from bottom to top: 0 to 500 ng mL^−1^); (b) linear equation between SWV current and the logarithm of the concentration of cTnI. Error bars: SD, *n* = 3.

Dynamic response range and sensitivity are both significant indexes to evaluate the analysis performance of the biosensor. Under the above optimization experimental parameters, we have also investigated the sensitivity of this sensor. The sensitivity and sensing performance of this sensing platform were studied with the concentration of cTnI from 0 to 500 ng mL^−1^. As shown in Fig. 4a, it can be clearly seen that with the increase in cTnI, the corresponding current response also shows a gradual increase, which indicates that the concentration of cTnI has a strong correlation with the current response. Simultaneously, Fig. 4b shows that there is a good linear correlation between the value of the current response and the logarithm of the concentration of cTnI in the linear range from 0.5 ng mL^−1^ to 100 ng mL^−1^. The regression equation of linear correlation was calculated as *i* = 2.1838 lg *C* − 4.3281 (*R*^2^ = 0.982), where *i* is the value of the SWV current response and lg *C* is the logarithm of the concentration of cTnI (ng mL^−1^). Also, the detection limit of this sensor platform can be as low as 40 pg mL^−1^ after calculation, which shows that the sensor has high sensitivity. In addition, we also compared this method with the other related literature reports listed in Table 2.

**Table 2:** Comparison of our strategy with other previously reported cTnI biosensors

Detection method	Detection limit	Linear range	Ref.
Silicon nanowire biosensors	0.005 ng mL^−1^	—	21
Fluoro-microbead guiding chip	1 ng mL^−1^	0.1–100 ng mL^−1^	14
Electrochemiluminescence immunosensors	0.002 ng mL^−1^	0.0025–10 ng mL^−1^	22
Optomagnetic biosensors	0.03 ng mL^−1^	0.03–6.5 ng mL^−1^	25
Nanoelectrode arrays	0.2 ng mL^−1^	0.1–100 ng mL^−1^	23
Electrochemical biosensors	0.04 ng mL^−1^	0.5–100 ng mL^−1^	This sensor

### 3.5. Specificity and regeneration

**Fig. 5:**
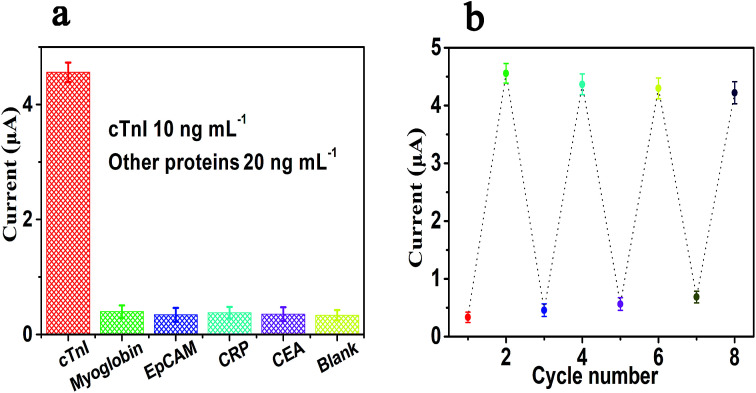
The specificity (a) and regeneration (b) of this electrochemical sensing platform for the detection of cTnI. Error bars: SD, *n* = 3.

Due to the specific recognition between cTnI and the aptamers of cTnI, the electrochemical sensing platform shows high specificity. There are numerous proteins in real biological samples, so we tested whether these proteins will interfere with the determination of cTnI. The specificity of this strategy for the determination of cTnI was also reviewed by adding five different kinds of proteins, which included cTnI and four other types of proteins, namely myoglobin, EpCAM, CRP, and CEA. As shown in Fig. 5a, we used this sensor to detect the other four kinds of proteins, namely myoglobin (20 ng mL^−1^), EpCAM (20 ng mL^−1^), CRP (20 ng mL^−1^), and CEA (20 ng mL^−1^); by comparing with the blank control group, we can see that there is no significant difference between the current response values of these four groups of proteins and the blank control group (the difference can be ignored). However, when we use this sensor to detect the value of the current response generated by cTnI (10 ng mL^−1^), we can see that the current response of cTnI is significantly stronger than any of the above four groups of proteins. In other words, the aforementioned results show that the sensing platform has an excellent specificity towards the detection of cTnI. Moreover, we have further studied the regeneration ability of the sensor; the experiment of regeneration was conducted by washing the sensor with a 5 M urea solution at 37 °C for 10 min. It can be seen from Fig. 5b that after five cycles of regeneration the value of the current response is almost at the same level as the first current response signal, indicating that the sensor has a satisfactory ability of regeneration.

### 3.6. Application in biological samples

**Fig. 6:**
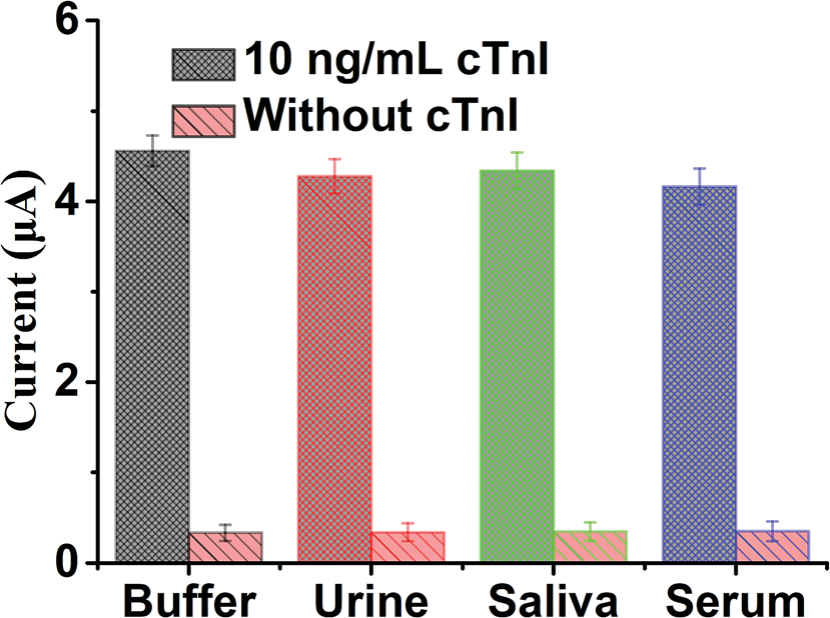
The SWV currents of electrochemical sensing platform for the detection of cTnI in several spiked biological samples, including urine, saliva and serum. Error bar: SD, *n* = 3.

In order to further study the selectivity and applicability of the sensing platform in various biological samples, we prepared three biological samples, namely human serum (10%), urine (10%) and saliva (10%), by diluting them. cTnI with the same volume and same concentration of 10 ng mL^−1^ was diluted to the above three biological samples and Tris buffer respectively. Also, each group was set with a blank control, and then the SWV current response values of each group were detected. The results in Fig. 6 show that in the presence of 10 ng mL^−1^ cTnI, the SWV current response of each group in different biological samples significantly enhanced in comparison with the blank control. The above results show that the sensing platform has excellent applicability and selectivity in biological samples, which is due to the specific recognition and high affinity of cTnI aptamer and cTnI. In addition, due to the excellent applicability and feasibility in biological samples of this sensing platform, this method also shows potential clinical practicability for the detection of cTnI.

## 4. Conclusion

In conclusion, we have successfully developed an electrochemical aptasensing platform for the detection of cTnI, which is based on the combination of highly specific recognition of aptamers with the TdT-mediated signal amplification strategy. The designed sensor has satisfactory specificity, sensitivity and regeneration, and the detection limit of this sensor can be as low as 40 pg mL^−1^. In addition, this method has been also applied to detect cTnI in various biological samples successfully, which shows that this method has a good potential for the clinical diagnosis, prognosis, and treatment of CVDs and AMI. Also, this method has universal applicability for the detection of other markers of disease. We only need to adjust the sequence of nucleic acid accordingly if the target to be detected is replaced by another marker. Compared with the antibody, the aptamer used in this study has good stability and low cost. Finally, the electrode used in this research can be regenerated, further reducing the cost. We believe that this sensing strategy will be a useful method to directly monitor other biomarkers by selecting appropriate aptamers, and will also provide potential references for the early detection of clinical biomarkers in the future.

## Conflicts of interest

There are no conflicts to declare.

## Supplementary Material
